# Potentials and Pitfalls of Cross-Translational Models of Cognitive Impairment

**DOI:** 10.3389/fnbeh.2019.00048

**Published:** 2019-03-14

**Authors:** Noor Z. Al Dahhan, Fernanda G. De Felice, Douglas P. Munoz

**Affiliations:** ^1^Centre for Neuroscience Studies, Queen’s University, Kingston, ON, Canada; ^2^Institute of Medical Biochemistry Leopoldo de Meis, Federal University of Rio de Janeiro, Rio de Janeiro, Brazil; ^3^Department of Psychiatry, Queen’s University, Kingston, ON, Canada; ^4^Department of Biomedical and Molecular Sciences, Queen’s University, Kingston, ON, Canada

**Keywords:** animal models, translational research, behavioral biomarkers, cognitive impairment, treatments

## Abstract

A number of clinical disorders that are either neurodevelopmental or neurodegenerative exhibit significant cognitive impairments that require some form of intervention. However, the current paucity of pro-cognitive treatments that are available, due to the lack of knowledge of biological targets and symptomologies, impedes the treatment of individuals with cognitive impairments. In this review article, we explore three critical steps that need to be established in order to lead to the development of effective and appropriate treatments for cognitive impairments. The *first* step specifically involves the ability to efficiently reproduce and standardize current animal models of disease. The *second* step involves establishing well-controlled and standardized animal models across different species, such as rodents and monkeys, that link to human disease conditions. The *third* step involves building these animal models from both a translational and a reverse translational perspective in order to gain critical insight into the etiologies of specific cognitive impairments and the development of their early physiological and behavioral biomarkers. This bidirectional translational approach is important to improve the investigation of disease biomarkers, the underlying mechanisms of novel therapeutics on cognition, and to validate preclinical findings of drug discovery. Overall, even though animal models play an important role in investigating the pathophysiological processes and mechanisms associated with typical and atypical behavior, we discuss the ongoing challenges associated with these three critical steps of cross-translational research that has led to the current lack of success of developing effective new compounds for potential treatments and suggest approaches to stimulate advances in the field.

Cognition involves complex processes that allow individuals to produce elegant thoughts and actions. These processes include attention, perception, planning, associative learning, working, episodic, and semantic memory, and language. Executive control processes, along with emotional and motivational processes, coordinate these functions in order to allow individuals to plan, choose, and regulate their behavior (Figure 1; Keeler and Robbins, 2011; Robbins, 2017). These cognitive processes are mediated by neural networks that range from specific systems to interactive cortical systems throughout the brain, including the frontal, occipital, parietal, temporal lobes, and subcortical structures. A number of clinical disorders that are either neurodevelopmental, such as attention deficit hyperactivity disorder (ADHD) and autism spectrum disorder (ASD), or neurodegenerative, such as Alzheimer’s disease (AD) and Parkinson’s disease (PD), exhibit significant cognitive impairments that require some form of intervention, such as pharmacological treatments.

**Figure 1:**
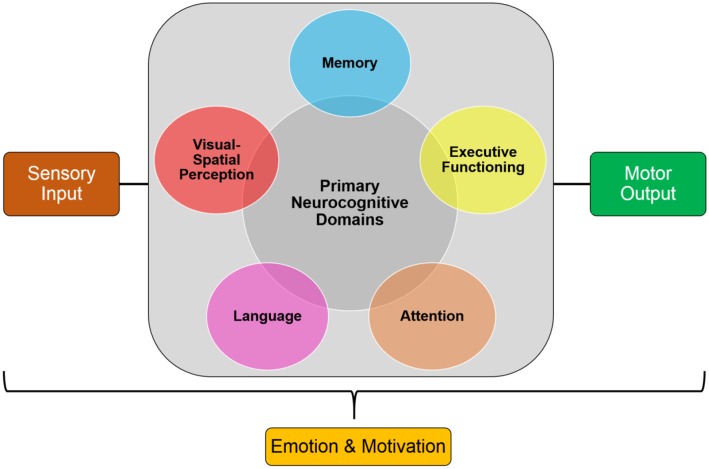
Simplified model of the core processes involved in cognition. These components can be used in cross-translational models to examine cognitive impairments in clinical disorders.

However, the current paucity of pro-cognitive treatments that are available, due to the lack of knowledge of biological targets and symptomologies, impedes the recovery of individuals with cognitive impairments (Homberg et al., 2016). These cognitive impairments can be extremely debilitating throughout an individual’s life: therefore, examining the underlying causes of these impairments is crucial to the development of effective and appropriate treatments and can change the functional outcomes and trajectories of these individuals (Cope et al., 2016). Translational neuroscience has become critical for developing preclinical models of cognitive impairments in these disorders in order to gain further insight into their etiologies and development, and to identify novel biomarkers and effective treatments (Figure 2; Keeler and Robbins, 2011). In addition to translational models, *reverse translational* models, in which patient-based findings guide the development of animal models, are equally as important to the development of early physiological and behavioral biomarkers of cognitive impairment in clinical disorders (Figure 2). These animal models typically comprise research involving *model organisms*, *test situations*, or *readouts*. For the purposes of this article, we refer to animal models in general that encompass these various research techniques. This bidirectional translational approach is important to improve the investigation of the underlying mechanisms of novel therapeutics on cognition, and to validate preclinical findings of drug discovery (Figure 2).

**Figure 2:**
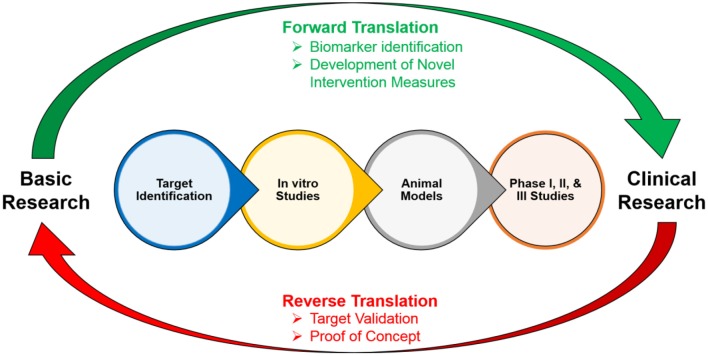
Cycle of the forward and reverse translation between basic and clinical research.

Even though animal models play a critical role in investigating the pathophysiological processes and mechanisms associated with typical and atypical behavior, there are a number of critical challenges that are associated with cross-translational research that has led to the current lack of success of potential treatments (De Felice and Munoz, 2016; Robbins, 2017). In this review we explore three critical steps that need to be established in order to lead to the development of effective and appropriate treatments for cognitive impairments: reproducing and standardizing animal models both: (1) within and (2) across species; and (3) building translational and reverse translational animal models of human disease conditions.

## Reproducing and Standardizing Animal Models

The *first* step that is necessary in order to lead to the development of effective and appropriate treatments for cognitive impairments specifically involves the ability to efficiently reproduce and standardize current animal models of disease. Animal models play an important role in investigating the pathophysiological processes and mechanisms associated with typical and atypical behavior, and translating these findings to the clinic by identifying novel pathways, biomarkers, and potential therapeutic interventions for treating clinical disorders (Matthews and Kopczynski, 2001; Snaith and Törnell, 2002; Porges, 2006; van der Staay et al., 2009; Nestler and Hyman, 2010; Belzung and Lemoine, 2011). Animal models that are designed to reflect a clinical disorder need to be both selective for a cognitive or behavioral variable that is relevant to that disorder, and also needs to simulate some component of that disorder in terms of its neural, molecular, or genetic component *via* a suitable intervention, such as a developmental, genetic, or an environmental neurotoxin (Keeler and Robbins, 2011). This underlying causality is typically replicated through mechanisms of action that are known from human disease conditions (McGonigle and Ruggeri, 2014). However, cognition is multi-faceted (Figure 1) and individuals can have impairments in either single or multiple processes. Thus, when developing an animal model of a disorder a major challenge is to target the entire spectrum of cognition (Kalueff et al., 2007; Kalueff and Stewart, 2015).

Therefore, a more reasonable strategy when designing an animal model is to deconstruct these disorders into simpler and easily quantifiable phenotypic units, or endophenotypes (Gottesman and Gould, 2003; Gould and Gottesman, 2006; van der Staay et al., 2009; Lenzenweger, 2013; De Felice and Munoz, 2016; Homberg et al., 2016). Animal models mimicking one or more symptoms of the cognitive impairments that are present in a clinical disorder can then be used to interpret the interactions that may be present between an induced deficit and the induced pharmacological models that are designed to remediate them (Homberg et al., 2016). However, a critical issue with this approach is that in most cases researchers are attempting to model a condition in animals that is still not well understood in humans. This creates a translational bottleneck in which the efficacy of a model becomes questioned.

### The Limitations of a Reductionist Model Approach: Clinical Heterogeneity

The challenge associated with the endophenotype strategy of creating animal models of a disease is that the focus of the model becomes narrow and phenotype-centered (Gottesman and Gould, 2003; Gould and Gottesman, 2006; Kalueff et al., 2008; Filiou et al., 2011; Ditzen et al., 2012; Lenzenweger, 2013; Maccarrone et al., 2013). While in many ways this is an advantage of a model because it allows researchers to accurately interpret interactions that may be present between an induced deficit and the induced pharmacological models that are designed to remediate them and facilitates the generalization of results from animal models to humans, it also serves as a critical disadvantage (van der Staay et al., 2009).

Clinical disorders are complex in nature and have multiple overlapping domains (Kas et al., 2007; Kalueff et al., 2008). For example, ASD is characterized by social and cognitive deficits, and behavioral perseverations. These deficits are not isolated, rather they are pathologically interlinked and form the core pathology of ASD. However, these deficits can also co-occur in an unrelated manner creating different disease phenotypes. For instance, combined high anxiety with cognitive deficits manifests as obsessive-compulsive disorder (OCD) and social phobia. Therefore, even though these disorders are similar clinically, they have different underlying neural pathogeneses (Robbins, 2017).

Furthermore, due to the multi-faceted nature of cognition, cognitive impairments vary within and between diagnoses (Cope et al., 2016). For example, the cognitive impairments that are present in an individual with early AD is different from the impairments that are present in an individual who has ADHD. However, there are commonalities in cognitive deficits that are found between disorders, such as those seen in AD and schizophrenia, that are likely due to overlapping underlying pathology of specific neural regions, such as the hippocampus (Keeler and Robbins, 2011). Therefore, the reductionist approach to assessing impairments in a disorder is complicated because different endophenotypes, or symptom clusters, can lead to the same impairment or diagnosis (Nestler and Hyman, 2010).

In addition to presenting the core symptoms of a clinical disorder, individuals also often present comorbid traits, such as altered sensory and/or emotional processing (Homberg et al., 2016). This creates an additional challenge of incorporating comorbid traits and assessing their associated symptoms when developing animal models of human disease conditions. Investigating comorbidity is important because the presence of these comorbid traits may interfere with specific behavioral responses during cognitive tasks, which becomes a confounding factor in the interpretation of the results of a model. However, this is also challenging because it is difficult to differentiate between comorbidity and disorder-specific domains (Kalueff et al., 2008).

In other words, if a clinical disorder consists of multiple distinct endophenotypes, then creating a preclinical and clinical model that examines and tests these different endophenotypes would overall be more accurate and valid compared to a model that specifically assesses only one phenotype. For example, when creating a model of ASD in which individuals have both social and cognitive deficits, it would be more accurate for a model to depict and assess both of these phenotypes and examine how they are related to one another as opposed to solely focusing on either the social or cognitive deficits (Silverman et al., 2010; Pearson et al., 2011; Reynolds et al., 2013; Kalueff and Stewart, 2015). However, the main challenge to this approach is that it becomes difficult to differentiate between the unique molecular mechanisms that are associated with either social deficits or cognitive deficits from those that are associated with their shared pathways (Kalueff and Stewart, 2015).

This is important to consider because it has been hypothesized that shared molecular and genetic pathways may underlie the pathogenetic coupling of specific endophenotypes, without individually affecting each endophenotype (Cope et al., 2016). For example, in ADHD, with which individuals present with both hyperactivity and attention deficits, there may be additional molecular mechanisms and networks that integrate these two endophenotypes resulting in individuals meeting the diagnostic criteria for ADHD. Therefore, the ability of an animal model to imitate both the disordered phenotypes and the interrelations between them, becomes an important challenge and consideration when developing experimental models of clinical disorders (Kalueff et al., 2008; LaPorte et al., 2010; Homberg et al., 2016; Robbins, 2017).

### The Influence of Genetic and Environmental Determinants

In addition to the complexity that is associated with cognition and cognitive impairments, clinical disorders typically have many environmental and genetic determinants that need to be taken into consideration because they may affect the success of preclinical and clinical cross-translational models (Jaffee and Price, 2007). For example, researchers have begun to examine the potential of early environmental interventions to reverse or rescue abnormal phenotypes in animal models (Nithianantharajah and Hannan, 2006; Morand-Beaulieu et al., 2015; Boyer et al., 2016). For instance, in a genetic model of Rett syndrome, mice that had a *MeCP2* deficit, but had early environmental interventions, led to a reversal of memory deficits, anxiety-like behavior, and motor coordination deficits (Lonetti et al., 2010). Similarly, in an animal model of fragile X syndrome, *Fmr1*-knockout mice that were raised in an enriched environment led to a reversal of both habituation deficits and hyperactivity (Restivo et al., 2005). These findings indicate the importance of examining both genetic and environmental determinants that may play important roles in the development of cognitive impairments in clinical disorders.

Furthermore, there are also genetic and environmental factors that need to be considered when making cross-translational comparisons. Rodents and non-human primates (NHPs) differ in both their genetic makeup and environmental exposure compared to humans (Searls, 2003; Martignoni et al., 2006). For example, the environment of an experimental animal is more homogenous in terms of social stimulus, temperature, fluids, and food compared to humans who have a heterogenous environment that may contribute to the expression of their impairments (McGonigle and Ruggeri, 2014). In addition to environment, age is different between animals and humans. Compared to humans, rodents have shorter life spans which makes the life expectancy shorter (~20 months) and redefines what is meant by chronic. For instance, in humans the onset of clinical signs of AD may take 10–20 years after the start of pathological processes, whereas aged animal models used for studying disease state cognitive impairments, such as mouse models, are 17–24 months old and transgenics are typically only weeks to months old (McGonigle and Ruggeri, 2014). While this makes it easier to study a disease process in an experimental setting, it should also be taken into consideration when examining results of a study. Overall, these results indicate that different environmental conditions can potentially affect the expression of a specific phenotype. Therefore, investigating gene-environment interactions and how this plays a role in animals and humans is important for translating findings to the clinic, and detecting potential environmental triggers that may play a confounding role in the expression of a behavioral trait (Nithianantharajah and Hannan, 2006).

One way to investigate these gene-environment interactions is through epigenetics, which examines how environmental factors change gene expression without altering primary DNA sequences (Rodenhiser and Mann, 2006). Epigenetic mechanisms of pathogenesis have been implicated in several neurodevelopmental and neurodegenerative central nervous system (CNS) disorders in which disruptions in learning and memory are the primary clinical symptom, such as Rett syndrome and AD (Day and Sweatt, 2011). Epigenetic animal models have been critical to not only understanding the cause and epigenetic effect associated among the risks of developing a disease, changes in DNA methylation, and alterations of specific environmental components, but also the long-term transgenerational epigenetic effects that are due to changes in the environment (Rosenfeld, 2010). Due to the prolonged nature of neurodevelopmental and neurodegenerative CNS disorders, a critical route for therapeutic interventions may be targeting the underlying epigenetic causes associated with a disorder. For example, rather than focusing interventions on targeting specific disease-causing gene mutations it may be more appropriate to focus on attempting to change the methylation status of specific candidate genes.

## Standardizing Animal Models Across Species

The *second* step that is necessary in order to lead to the development of effective and appropriate treatments for cognitive impairments involves establishing well-controlled and standardized animal models across different species, such as rodents and monkeys, that link to human disease conditions.

### Cross-Species Tasks of Cognitive Function

To develop targeted pro-cognitive compounds in patients with clinical disorders, researchers need to first understand the underlying neural mechanisms associated with cognitive impairments. These cognitive domains need to be similarly measured in humans and animals to efficiently test novel targets before clinical trials are conducted in order to avoid translational bottlenecks (Young and Geyer, 2015). This is important because quantifying cognitive impairments using tasks that have cross-translational validity would not only allow for the quantification of comparable processes *among* species, but will also increase the probability that the neural substrates underlying these processes are conserved *across* species. This in turn will bridge the current gap between preclinical and clinical testing, and will lead to the possibility of developing effective treatments for cognitive impairments (Figure 2; Young and Geyer, 2015).

One of the biggest challenges of studying processes that are involved in cognition is that it is largely multi-faceted and componential in nature which makes it difficult to compare cross-translational performance during specific tasks (Figure 1). For example, disorders in which there are deficits in attention and/or motivation, as found in ADHD and schizophrenia, may lead to secondary deficits in memory, decision making, and inhibitory control. This illustrates that in order to develop cross-species tests that examines processes involved in memory, it is essential to first administer tests that examine basic behavioral controls, such as attention, sensory, motor and/or motivational factors, to ensure that there are no secondary or indirect confounding factors contributing to the results that are obtained (Keeler and Robbins, 2011). Therefore, a broader characterization of animal models across multiple cognitive domains and tasks will further the understanding of the consequences of specific etiological manipulations. In addition, administering tasks that have translational validity would improve attempts to create novel therapeutics for patients with impairments in those cognitive domains (Bussey et al., 2013; Gilmour et al., 2013; Keeler and Robbins, 2011; Young and Geyer, 2015). This creates a broader picture of the underlying mechanisms resulting in cognitive impairments in clinical disorders which leads to the ability to develop more targeted therapeutics.

Cross-translational models of cognitive impairment need to assess performance during cognitive-behavioral tasks that have construct validity (Cope et al., 2016). Despite a number of animal models that have been developed to examine cognitive impairment in clinical disorders, there are a number of limitations and caveats to the interpretations of these studies. This is because the cognitive tests that were used were relatively narrow in scope due to the use of specific diagnostic criteria that were used to develop animal models *ad hoc*. For example, if a disorder is characterized by deficits in working memory, researchers have attempted to model and reproduce these deficits in experimental animals. However, a critical issue with this approach is that researchers are neglecting the influence other domains may be having due to the experimental manipulation (Cope et al., 2016). For example, impairments in spatial navigation due to amnesic drugs can be confounded by effects on motor ability, or if animals display reduced motivation to work for rewards this would in turn negatively impact their performance on reward-based tasks. Furthermore, there may be cross-species similarities or differences in neural mechanisms sub serving specific cognitive behaviors. Therefore, it is important to create a complete profile of cognitive impairments across multiple domains in order to examine the influence of multiple processes, and to also assess cross-species similarities in neural activation during cognitive tasks to examine similarities/differences in underlying neural mechanisms.

To address this, studies using animal models should administer a wide battery of tests in order to not only determine the profile of cognitive deficits that may be present, but to also examine whether improvements and/or deficits in one domain are related to improvements and/or deficits in another domain (Keeler and Robbins, 2011). Furthermore, these batteries of tests should balance those that examine learning with those that examine performance. When an animal is trained on a specific task, this eventually leads to relatively stable task performance baselines. These baselines can then be used to examine any perturbations that are produced by pharmacological challenges and/or by dose-related therapeutic comparisons.

Some studies have developed cognitive test batteries that are very similar to those employed in human studies, using equipment and tasks that are also similar to those employed in human studies. One such platform for this type of translation is the Cambridge Neuropsychological Test Automated Battery (CANTAB) which can be used to probe cognitive performance on different tasks that probe aspects of visuospatial learning, spatial working memory, impulse control, discrimination learning, and skilled motor performance. Such standardized batteries have been validated for work in NHPs (e.g., Weed et al., 1999; Nagahara et al., 2010) and rodents (e.g., Bussey et al., 2012; Hvoslef-Eide et al., 2016; Creighton et al., 2019). While previous studies have successfully used automated cognitive testing in NHPs (e.g., Bartus et al., 1978; Voytko, 1993; Buccafusco et al., 2002; Moore et al., 2005), the CANTAB set of tasks utilizes the same testing hardware and comparable software across species, potentially supporting more direct comparisons of age-related deficits across species. Establishing similarities of cognitive decline in aged monkeys and humans would demonstrate cross-species effects of aging and substantiate the potential relevance and importance of NHP studies in understanding changes in neuronal substrates that underlie human aging. Furthermore, cognitive testing in the mouse is now getting more and more stereotyped and validated. For example, Mousebytes^1^ is an open access database for mouse cognition, imaging, and genomics data integration. Cognitive testing with a diverse battery of tasks can reveal dissociable impairments in different strains of AD mice (Creighton et al., 2019).

## Building Translational and Reverse Translational Animal Models

Lastly, the *third* critical step involves building these animal models from both a translational and reverse translational perspective in order to gain critical insight into the etiologies of specific cognitive impairments and the development of their early physiological and behavioral biomarkers.

### The Challenge of Validity

The lack of translational biomarkers and treatments from animal models to clinics has emphasized the importance of having a framework of validity that is used when assessing the efficacy of an animal model (van der Staay et al., 2009; Nestler and Hyman, 2010; McGonigle, 2014; McGonigle and Ruggeri, 2014; Stewart and Kalueff, 2015). The most common types of validity that are assessed are: (a) *construct validity*, the degree to which animal models conform to and embody etiological aspects of a disorder; (b) *face validity*, the extent to which animal models reflect the main symptomology or features of a disorder; and (c) *predictive validity*, the applicability of a model in predicting clinically effective therapeutic agents (Kalueff et al., 2007; Stewart and Kalueff, 2015). Even though these criteria encourage a careful and systematic evaluation when developing animal models of disease, these criteria are relatively hard to meet (McGonigle and Ruggeri, 2014). The main reason for this is because for several clinical disorders, such as AD or PD, the underlying etiology of the disorder is not well understood which affects the ability to establish all of these criteria.

Construct validity is argued to be the most important criteria when examining an animal model because it is thought to reflect the soundness of the theory that underlies a model and provides a framework for interpreting the data that is produced (van der Staay et al., 2009). Ideally, high construct validity would be achieved in an animal model by replicating the behavioral and neural characteristics of a disorder, as well as recreating the etiological processes that lead to an impairment (Chadman et al., 2009). This can be accomplished by: genetic manipulations, altering the function of specific pathways or neural circuits that are hypothesized to be involved in a disorder’s pathogenesis, or by exposing an animal to a known disease-causing agent or well-validated environmental risk factor (Nestler and Hyman, 2010). However, the challenge with this approach is differentiating whether results, in the absence of relevant human genetic evidence, represents interesting phenocopies of a disease or whether they represent a legitimate disease model with useful implications for developing therapeutics (Nestler and Hyman, 2010).

Face validity is the similarity between cognitive impairments observed in an animal model and in humans that are affected by a disorder, such as AD (van der Staay et al., 2009). However, the emphasis on having high face validity in a model has been criticized, because the same cognitive impairments that are present in both animals and humans may be the result of different underlying mechanisms, or similar deficits could serve different functions (Sarter and Bruno, 2002; Sarter, 2004; van der Staay et al., 2009). In addition, creating a strong emphasis on having high face validity in a model may create an obstacle for developing animal models with phylogenetically lower species, because there is a greater degree of similarity of symptoms in species that are phylogenetically closer to humans (van der Staay et al., 2009). Furthermore, it is unlikely that specific behaviors will precisely model human situations, or animal models will recapitulate all the behavioral features that are observed in humans (Nestler and Hyman, 2010). This similarly relates to the issue of placing a strong emphasis on predictive validity because some clinical disorders have poor therapeutic standards which leads to only a few weakly effective compounds that can be clinically used. This approach then results in an animal model being deemed unsuitable to detect novel therapeutic interventions (Sarter, 2004).

To address the limitations of these criteria, preclinical paradigms should form two groups. With one group assessing phenotypes that are linked to clinical disorders through behavioral tests, and have high predictive and face validity, and the other group assessing a disorder’s pathogenesis through experimental models, and thus has high construct validity (Homberg et al., 2016). These two groups should be used in parallel with one another in order to gain further insight into the underlying mechanisms contributing to the development of cognitive deficits in a clinical disorder, and to increase the translational prospects of a model.

### Translation of Animals to Humans and Back Again

To create an ideal animal model of a disorder, it needs to have the following features: similar anatomy and physiology, similar genetic basis, similar pathological response and underlying etiology, similar phenotypic endpoints as clinical studies, responsive to known treatments with clinical efficacy, and has to be predictive of clinical efficacy. The practical attainability of many of these features is debatable, but a systematic assessment of how an experimental model fares in regard to these features provides valuable insight regarding the strengths and weaknesses of a model and how it may be used (McGonigle and Ruggeri, 2014).

In order to improve and further the understanding of cognitive impairments that are present in a majority of clinical disorders, it is important to create a complex, systems biological-based approach which parallels preclinical animal models with clinical studies and mechanistically-driven *in vitro* research (Kalueff et al., 2007; Kalueff and Stewart, 2015). Even though rodent models are valuable tools to study cognitive impairments in clinical disorders, it is important to note that a majority of these deficits involve higher cognitive functions which are hard to investigate in detail in rodents. For this reason, other animal models, such as NHP models based on the common marmoset (*Callithrix jacchus*) have gained popularity as a tool to study higher cognitive function impairments (Homberg et al., 2016). Humans and NHPs share a number of similarities in terms of the organization of functional networks and overall neural architecture and is a good candidate to examine cognitive impairments in humans and the underlying neural mechanisms that are involved. Therefore, a multi-disciplinary approach of investigating cognitive impairments in both rodents and NHP models is important to examine how these deficits develop in the brain, and ways to maximize the success of therapeutic interventions and remediations (De Felice and Munoz, 2016).

There have been efforts in the field to develop NHP models of human diseases with the ultimate goal to improve translational efficacy of potential therapeutics. For example, NHP models of autism have been developed to examine the link between autism-related genes, such as *MeCP2*, and disease symptomology (e.g., Liu et al., 2014, 2016). These studies have found that compared to wild-type monkeys, *MeCP2* transgenic monkeys exhibited signs of stereotypic disease behavior, such as: a higher frequency of repetitive circular locomotion, increased stress responses, and less interaction with other wild-type and transgenic monkeys in social interaction tests (Liu et al., 2014). Furthermore, NHP models of AD have also been developed. In a study using cynomolgus NHPs, key pathological features of AD were induced when Aβ oligomers, AD-related toxins, were injected into the lateral ventricle (Forny-Germano et al., 2014; Batista et al., 2018). Aβ oligomers were found to diffuse into the NHP brain and accumulated in several regions, including frontal cortex, hippocampus, and amygdala. After sacrifice, cardinal features of AD pathology, including synapse loss, tau hyperphosphorylation, tangle formation and neuroinflammation were observed in regions of the macaque brain where Aβ oligomers were abundantly detected. These findings illustrate that refining NHP models of human diseases is crucial to developing translational research. The next step is to quantify in detail the cognitive impairments produced in these animals, how they relate to the underlying pathophysiology, and ultimately how it relates to the cognitive impairments in humans with AD.

In addition to translational models, *reverse translational* models are equally as important to the development of early physiological and behavioral biomarkers of cognitive impairment in clinical disorders (Figure 2; Homberg et al., 2016). For example, multivariate analyses of neurological development comparing typically developing infants and those with abnormal neural developments have found significant correlations between specific behavioral phenotypes, such as rolling behavior and head control, with physiological biomarkers, such as neural imaging data (Koshiba et al., 2015). This indicates that behavioral phenotypes can potentially be used as a biomarker for the development of specific disorders in the absence of more complex behaviors. This in turn would be especially important to the future functional outcomes and trajectories of an individual because there is a critical window in early neural development for maximizing the success of remediations and interventions (Cope et al., 2016).

Overall, this bidirectional translational approach is important to improve the investigation of the underlying mechanisms of novel therapeutics on cognition, and to validate preclinical findings of drug discovery. In this approach, proof of principle studies in healthy and human volunteers are conducted to test the effects of pro-cognitive treatments with known safety profiles, and parallel studies in experimental animals are conducted to define the underlying neural mechanisms of the therapeutic (Figure 2; Robbins, 2017). However, this bidirectional approach relies on the ability to administer efficient cross-translational cognitive measures so that informative comparisons can be made across species.

## Conclusions

Animal models of cognitive impairment have played an important role in furthering the understanding of the underlying pathology and molecular mechanisms of clinical disorders and evaluating the efficacy of potential therapeutic interventions (Robbins, 2017). Despite these potentials, there are a number of challenges associated with cross-translational research that has led to the current lack of success of developing effective new compounds of potential treatments. Due to the large expense associated with Phase 3 clinical trials, this lack of success has led to the questioning of the use of animal models to study cognitive impairments in clinical disorders. To address this, in this article three critical steps that are needed to lead to the development of effective and appropriate treatments for cognitive impairments were explored: reproducing and standardizing animal models both: (1) within and (2) across species; and (3) building translational and reverse translational animal models of human disease conditions.

Overall, one of the largest cross-translational challenges of developing animal models is the inability to mimic all the symptoms and underlying neural mechanisms that are present in these disorders and the vast heterogeneity that is present in clinical disorders and patient groups. This is largely due to the fact that the cognitive impairments that are present in these disorders arise from a complex interplay of genetic, environmental, developmental, and neural abnormalities that cannot all be sufficiently modeled in experimental animals. However, despite these limitations, experimental animal models are crucial and necessary to advance the understanding of the cognitive impairments that underlie a majority of clinical disorders, and to study behavioral, gene expression patterns, and neuronal morphology that are present before and during the manifestation of a disorder (Homberg et al., 2016). Furthermore, preclinical models allow researchers to assess the functional impact of genes and gene networks at the level of neuronal, synaptic, and genetic networks of cognition, behavior, and neural systems. Therefore, paralleling both clinical findings with animal models will lead to more mechanistic and translational insights into the pathogenesis of clinical disorders.

## Author Contributions

NA, FDF, and DM wrote the article.

## Conflict of Interest Statement

The authors declare that the research was conducted in the absence of any commercial or financial relationships that could be construed as a potential conflict of interest.

## Footnotes

^1^mousebytes.ca
